# The 3-triangle method preserves the posterior tibial slope during high tibial valgus osteotomy: first preliminary data using a mathematical model

**DOI:** 10.1186/s40634-022-00466-y

**Published:** 2022-03-23

**Authors:** Manuel Weißenberger, Mike Wagenbrenner, Fritz Schote, Konstantin Horas, Thomas Schäfer, Maximilian Rudert, Thomas Barthel, Tizian Heinz, Stephan Reppenhagen

**Affiliations:** 1 Department of Orthopaedic Surgery, University of Wuerzburg, Koenig-Ludwig-Haus, Brettreichstr. 11, 97074 Wuerzburg, Germany; 2 Department of Orthopaedic Surgery and Traumatology, University of Salzburg, University Hospital Salzburg SALK, Müllner Haupstraße 48, 5020 Salzburg, Austria

**Keywords:** Knee, High tibial valgus osteotomy, Axial alignment, Posterior tibial slope, Weight bearing line, Cartilage, Triangle method, Osteoarthritis

## Abstract

**Purpose:**

Despite much improved preoperative planning techniques accurate intraoperative assessment of the high tibial valgus osteotomy (HTO) remains challenging and often results in coronal over- and under-corrections as well as unintended changes of the posterior tibial slope. Noyes et al. reported a novel method for accurate intraoperative coronal and sagittal alignment correction based on a three-dimensional mathematical model. This is the first study examining preliminary data via the proposed Noyes approach for accurate intraoperative coronal and sagittal alignment correction during HTO.

**Methods:**

From 2016 to 2020 a total of 24 patients (27 knees) underwent HTO applying the proposed Noyes method (Noyes-Group). Radiographic data was analyzed retrospectively and matched to patients that underwent HTO using the conventional method, i.e., gradual medial opening using a bone spreader under fluoroscopic control (Conventional-Group). All operative procedures were performed by an experienced surgeon at a single orthopaedic university center.

**Results:**

From the preoperative to the postoperative visit no statistically significant changes of the posterior tibial slope were noted in the Noyes-Group compared to a significant increase in the Conventional-Group (*p* = 0.01). Regarding the axial alignment no significant differences between both groups were observed pre- and postoperatively. The number of over- and under-corrections did not differ significantly between both groups. Linear regression analysis showed a significant correlation of the postoperative medial proximal tibial angle (MPTA) with the position of the weightbearing line on the tibial plateau.

**Conclusion:**

The 3-triangle method by Noyes seems to be a promising approach for preservation of the posterior tibial slope during HTO.

## Introduction

Osteoarthritis of the knee joint is a well-known musculoskeletal disorder which especially affects the elderly population. Arthroplasty surgery has been proven to be an effective method for alleviating pain and restoring knee function, especially in the functionally low-demand and older patients. However, less favorable results for total knee arthroplasty (TKA) have been shown in a younger and functionally demanding patient collective [3], calling for alternative treatment strategies such as the high tibial osteotomy (HTO). Therefore, amid the clinical success and popularity of TKA, HTO remains an indispensable treatment choice for the young and active patient with isolated and symptomatic osteoarthritis of the medial knee compartment [4, 12]. Furthermore, recent advances in plate fixation, surgical techniques, appropriate patient selection and preoperative planning have further improved results following HTO [9, 22]. However, durability and longevity of the performed HTO have been an ongoing issue ever since [21, 24]. Precise preoperative planning of the intended valgus correction and meticulous intraoperative realignment according to the preoperatively determined values have been revealed as a key factor in terms of patient satisfaction and clinical success of the HTO [8, 11, 22]. In addition, ongoing controversy regarding the ideally desired postoperative alignment remains. Most authors aim for a valgus overcorrection of about three degrees with the weightbearing line intersecting the tibial plateau at about 62% of the tibial plateau width (measured from the medial to the lateral edge), which roughly corresponds to the lateral tibial spine [10, 13, 21, 22]. The methods to achieve and control the coronal angular correction intraoperatively vary widely, reaching from conventional methods through preoperative templates and intraoperative gap measurement to intraoperative computer assisted navigation systems. Preoperatively obtained full-length lower limb weightbearing radiography has become the standard method for planning of the desired valgus correction according to Dugdale et al. and Miniaci et.al [6, 18]. A new algebraic-based method by Noyes et al. promises proper angular correction by various intraoperatively obtained gap measurements along the osteotomy site and preoperatively obtained long-leg radiographs [19]. The aim of this study was to verify the accuracy of this mathematical model in an everyday clinical setting. It was hypothesized that the aforementioned 3-triangle method by Noyes et al. would allow for a more accurate coronal and sagittal correction compared to the conventional correction osteotomy planning using landmark-based digital software such as mediCAD (Hectec GmbH, Germany) [19].

## Materials and methods

### Study design and subjects

This retrospective study was conducted at a single orthopaedic center of a university department and was approved by the local ethics committee of the university. A total of 54 knees from 51 patients were analyzed retrospectively. From 2016 to 2020 a total of 24 patients (26 knees) underwent open wedge HTO for medial compartment knee osteoarthritis and varus malalignment using the correction planning technique described by Noyes et al. (Noyes-Group). Patients from the Noyes-Group were matched to a control group using the conventional osteotomy planning technique by landmark-based digital software (mediCAD, Hertec Gmbh, Germany) (Conventional-Group). Correction planning using landmark-based software was considered the gold standard at the orthopaedic center. Patients from the Conventional-Group underwent HTO from 2006 to 2012 for medial knee osteoarthritis and varus malalignment (total 26 patients). Propensity score matching was performed and individuals from the Noyes-Group were assigned by nearest neighbor-matching to individuals from the Conventional-Group based on similar propensity scores. Age, BMI and angular deformity parameters (WBL, JLCA, MPTA, PTS) were set as covariates in the matching process. Demographics and surgical data of both groups are displayed in Table 1. Inclusion criteria were defined as follows: 1) Age < 65 years, 2) body mass index (BMI) < 40, 3) isolated medial joint pain, 4) high level of activities (except jumping and running), 5) knee flexion ≥90 degrees, 6) absence of flexion contracture of the knee > 5°. Patients who did not meet the inclusion criteria or individuals with bi- or tricompartimental osteoarthritis of the knee were excluded from HTO. Furthermore, an intact ACL (anterior cruciate ligament) was defined as a requirement for inclusion. For both groups postoperative radiographs were obtained at 6 weeks after the operative procedure.

**Table 1:** Demographics and surgical data

Variable	Noyes-Group	Conventional-Group	***P***-value
**Age (years)**	43.7 ± 12.1	43.3 ± 11.7	0.91
**BMI (kg / m**^**2**^**)**	26.9 ± 5.5	26.1 ± 4.7	0.58
**Sex (male / female)**	4 / 23	5 / 22	0.50
**MPTA (°)**			
**Preoperative**	84.2 ± 2.8	84.5 ± 2.6	0.70
**Postoperative**	90.4 ± 2.7	92.4 ± 3.3	0.03
**Weight bearing line (%)**			
**Preoperative**	19.3 ± 10.1	17.2 ± 7.5	0.39
**Postoperative**	48.1 ± 12.2	53.3 ± 15.7	0.19

Demographics and surgical data of both groups. Values are presented as mean ± standard deviation

*MPTA* Medial proximal tibial angle

### Preoperative planning

Long-leg weightbearing radiographies of the affected extremity were obtained preoperatively in two planes from all patients undergoing HTO. All radiographies were captured and processed digitally using PACS (Picture Archiving and Communication System). The weightbearing line (WBL), defined as a line from the center of the femoral head to the center of the ankle joint was determined and its intersection at the tibial plateau was expressed as percentage of the total tibial width according to Dugdale et al. [6]. Coronal angular valgus correction was determined using digital planning software (mediCAD, Hectec Gmbh, Germany). Target parameters for valgus correction were defined as follows: 1) Intersection of the weightbearing line at 62.5% of the tibial plateau width, which transfers approximately to the lateral tibial spine, 2) a mechanical femorotibial angle of 3 to 5 degrees of valgus. Furthermore, the medial proximal tibial angle (MPTA) and mechanical lateral distal femur angle (mLDFA) were obtained routinely in order to assess the location of the varus deformity ruling out a combined femoral and tibial varus deformation. Six weeks after the surgery another full length weightbearing long-leg and lateral knee X-rays was obtained to check postoperative alignment and proper plate fixation. Posterior tibial slope (PTS) was measured on true lateral knee radiographies according to the method described by Brazier et al. [2]. The PTS angle was defined as the angle between the tangent of the medial tibial plateau and a line perpendicular to the posterior tibial cortex. All radiographic measurements were performed by two orthopedic trained physicians and an intraclass-correlation coefficient was calculated (ICC = 0.68).

### Surgical techniques

**Fig. 1:**
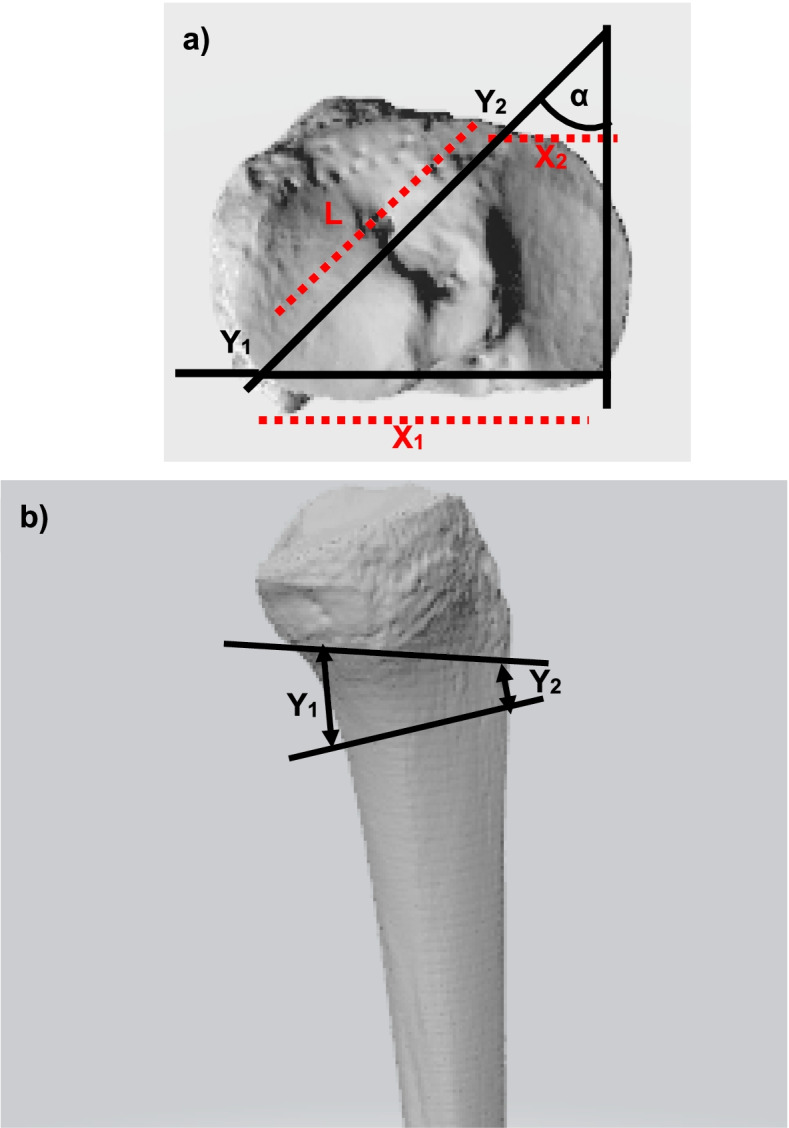
**a** Axial view of the tibial plateau: The height of the vertical gap at the posteromedial osteotomy site (Y_1_) is a function based on the law of triangles. Subsequently, the height of the vertical gap at the anteromedial osteotomy site (Y_2_) is determined based on the distance L between the two gap measurements, the height of the vertical gap measurement at Y_2_, the tibial width X_1_ and the angle of the anteromedial tibial cortex α. **b** Sagittal view of the proximal tibia: In case of equal anterior and posterior vertical gap height an increase of the posterior tibial slope will be observed. To maintain the normal posterior tibial slope the vertical gap height at the posteromedial cortex (Y_1_) will always be greater than at the anteromedial tibial cortex (Y_2_). Modified figure. Human Tibia (https://skfb.ly/6ursI) by Eric Bauer is licensed under Creative Commons Attribution (http://creativecommons.org/licenses/by/4.0/)

Surgical techniques did not differ between both groups and were performed by the same two senior surgeons. A biplanar L-shaped medial osteotomy was performed for both groups. In the conventional-Group the posteromedial osteotomy side was gradually opened using chisels and a bone spreader until the desired and preoperatively determined correction angle was achieved. Intraoperatively, the correction angle was checked using fluoroscopy while applying axial load to the foot sole. A sterile ruler was used to determine the intraoperative gap width at the posteromedial border. The exact same approach and osteotomy technique was used in the Noyes- and the Conventional-Groups. However, instead of opening the osteotomy under fluoroscopic control until achieving the desired correction angle in the coronal plane, the medial osteotomy side was gradually opened by a predefined amount at two points along the medial osteotomy side. The measurements of the wedge opening were determined intraoperatively based on the algebraic model of Noyes (Table 2, Fig. 1). Table 3 summarizes the steps for determining the gap width at the osteotomy site in detail. In every case, the opening wedge was secured using a TomoFix plate (Synthes, Solothan, Swiss). In cases of an osteotomy gap width > 15 mm or in cases of high non-union risk, gap augmenting with autologous bone or bone substitute material (ChroNos®) was performed (10 cases in total). The postoperative rehabilitation protocol did not differ for both groups and encompassed a six-week period of non-weightbearing ambulation and continuous passive motion exercises. A braced hinge allowing the knee-flexion up to 90 degrees was further applied for 6 weeks. After radiographic controls were obtained 6 weeks postoperatively progressive weightbearing was commenced and knee flexion was no longer limited.

**Table 2:** Osteotomy gap width at the anteromedial and posteromedial osteotomy site

Patient ID	OT gap at Y_1_	OT gap at Y_2_	Tibial width at Y1	Length Y_1_ to Y_2_
1	8,0	6,8	50	10
2	13,0	10,0	60	20
3	9,0	6,5	55	20
4	9,0	6,5	50	20
5	11,0	9,0	60	10
6	9,0	6,5	50	20
7	8,0	6,0	55	20
8	10,0	7,5	60	20
9	9,0	7,5	60	15
10	10,0	7,5	55	20
11	10,0	8,0	55	20
12	11,0	7,0	65	20
13	12,0	9,0	55	20
14	11,0	7,0	60	20
15	11,0	8,5	60	20
16	11,0	8,5	60	20
17	10,0	7,5	60	20
18	11,0	8,5	55	20
19	9,0	7,5	55	20
20	13,0	9,0	70	25
21	10,0	7,2	60	20
22	12,0	9,0	70	25
23	9,0	6,0	65	20
24	6,0	4,5	60	20
25	10,5	8,0	65	20
26	8,0	6,0	60	20
27	5,0	4,0	65	20

Osteotomy (OT) gap width at the anteromedial and posteromedial osteotomy site in mm as calculated intraoperatively using the mathematical model according to Noyes

**Table 3:** Intraoperative steps for proper vertical gap opening at the osteotomy site

Steps for determining the gap width at the osteotomy site based on the algebraic model of Noyes	
1. The desired angular valgus correction was determined based on preoperative weightbearing long leg radiographs.	
2. Intraoperatively a controlled opening of the osteotomy was performed by measuring the gap width at two predefined points along the medial osteotomy site.	
3. The first vertical gap width measurement was performed at the most medial point of the tibia, i.e. the most posteromedial point of the osteotomy. The vertical gap width at this point was assigned by the letter Y_1_.	
4. To determine the vertical gap width Y_1_, the coronal tibial width was measured at the most posteromedial site of the osteotomy. The tibial width at this point is assigned by the letter X_1_. Based on the tibial width X_1_ and the preoperatively determined coronal valgus correction angle the vertical gap width at Y_1_ is calculated and the osteotomy was gradually opened until the calculated vertical gap width at Y_1_ was reached.	
5. Another vertical gap measurement was performed at a more anteriorly located point along the medial osteotomy site, assigned by the letter Y_2_. The distance of the two gap measurement points Y_2_ and Y_1_ along the medial osteotomy site was measured, assigned L. Furthermore, the coronal tibial width at Y_2_ was measured, assigned X_2_. From the tibial width X_2_, the vertical gap width Y_1_ and the distance L the vertical gap width Y_2_ was calculated and the osteotomy is gradually opened at Y_2_ until the calculated vertical gap width was reached.	

Performed intraoperative steps for obtaining proper vertical gap opening at the osteotomy site according to the algebraic method proposed by Noyes.

### Statistical analysis

Pre- and postoperative data were compared for both groups. Numerical data was analyzed using a two-sample t-test with an alpha level selected at 0.05 for statistical significance. Categorical variables were compared using Chi-square analysis. A logistic regression was used to evaluate a potential relationship between the preoperative varus malalignment severity and the outcome of alignment correction. All statistical analyses were performed using SPSS version 26 (IBM Co., Armonk, NY, USA). A posthoc power analysis was performed based on the selected sample size of the patients, alpha level (*p* = 0.05) and an estimated moderate effect size (d = 0.03) and turned out to be 0.78.

## Results

Regarding the posterior tibial slope, no worthwhile differences between the Noyes and Conventional-Group were noted at the preoperative visit (6.2° ± 4.0° versus 8.9° ± 4.2°). However, from the preoperative to the postoperative visit, a statistically significant increase was observed only within the Conventional-Group (*p* = 0.01) (Table 4). The PTS did not increase statistically significant in the Noyes-Group from the preoperative to the postoperative visit (Table 4). There was also statistically significant disparity in the mean values of the posterior tibial slope between the Conventional-Group and Noyes-Group at the postoperative visit (Table 4).

**Table 4:** PTS for the Noyes- 472 and conventional-group

Variable	Noyes-Group	Conventional-Group	***P***-value (comparison between both groups)
**PTS preoperative (mean ± SD)**	6.2° ± 4.0°	8.9° ± 4.2°	0.08
**PTS postoperative (mean ± SD)**	7.7° ± 4.6°	11.0° ± 5.0°	0.02*
***P*-value (comparison within a group)**	0.12	0.01*	

Preoperative and postoperative mean values of the posterior tibial slope (PTS) for the Noyes- and Conventional-Group respectively. *P*-values are displayed for comparison of the mean PTS from the pre- to postoperative visit within a group (last row) as well as for comparison of the PTS between the groups (last column). Statistically significant values are marked by asterisks

**Fig. 2:**
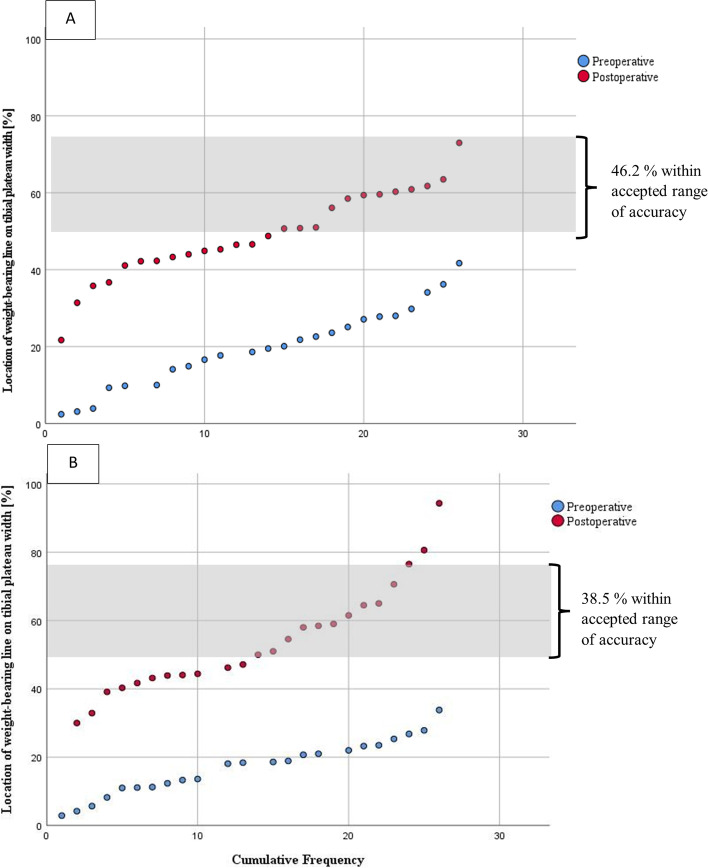
Cumulative frequency of the pre- and postoperative weight-bearing lines for the Noyes group (**A**) and Conventional-Group (**B**). The bar represents the accepted range of accuracy which corresponds to the 50–75% area of the tibial plateau width

Regarding the measurements of the weight-bearing line (WBL) no statistically significant differences were revealed during the preoperative visit. In the Noyes-Group the WBL crossed the tibial plateau at the mean 19.5 ± 10.2% coordinate of the medial to lateral coronal tibial width compared to the 17.3 ± 7.6% coordinate of the Conventional-Group (*p* = 0.39). Postoperatively, the mean location of weight bearing lines on the tibial plateau did not differ significantly between both groups (Noyes-Group: 49.1 ± 11.4%, range from 22% to 73%; Conventional-Group: 52.8 ± 15.7%, range from 30% to 94%, *p* = 0.33) (Table 5). However, there was a statistically significant lateral shift of the WBL within each group from the preoperative to the postoperative visit (Table 5). From the preoperative to the postoperative visit the mean location of the weight-bearing line was shifted about 30.0 ± 9.6% laterally along the coronal tibial plateau width in the Noyes-Group. In the Conventional-Group the weight-bearing line crossing was moved to a mean 35.6 ± 16.7% more lateral position along the coronal tibial plateau width. Comparison of the mean locational shift of the WBL between both groups did not reveal any significant differences (*p* = 0.15). In this study, the placement of WBL between the 50% and 75% coordinate of the coronal tibial plateau width ratio was set as the accepted range of accuracy. In the Noyes-Group 46.2% (12/26) of the patients were corrected within the accepted range of accuracy compared to 38.5% (10/26) in the Conventional-Group (Fig. 2). This difference turned out not to be of statistical significance (*p* = 0.58). Regarding the Noyes-Group 53.8% of patients were classified as under-corrected but no case of overcorrection was noticed. In the Conventional-Group 50.0% of patients have been under-corrected and another 11.5% haven been over-corrected according to the tibial crossing of the weight-bearing line.

**Table 5:** Location of the WBL for the Noyes- and Conventional-Group

Variable	Noyes-Group	Conventional-Group	***P***-value (comparison between both groups)
**WBL preoperative (mean ± SD)**	19.5 ± 10.2%	17.3 ± 7.6%	0.39
**WBL postoperative (mean ± SD)**	49.1 ± 11.4%	52.8 ± 15.7%	0.33
***P*-value (comparison within a group)**	0.00*	0.00*	

The location of the weight-bearing line (WBL) on the tibial plateau according to its medial to lateral total tibial width ratio is shown for the pre- and postoperative state. *P*-values are displayed for comparison of the mean WBL from the pre- to postoperative visit within a group (last row) as well as for comparison of the WBL between both groups (last column). Statistically significant values are marked by asterisks

**Fig. 3:**
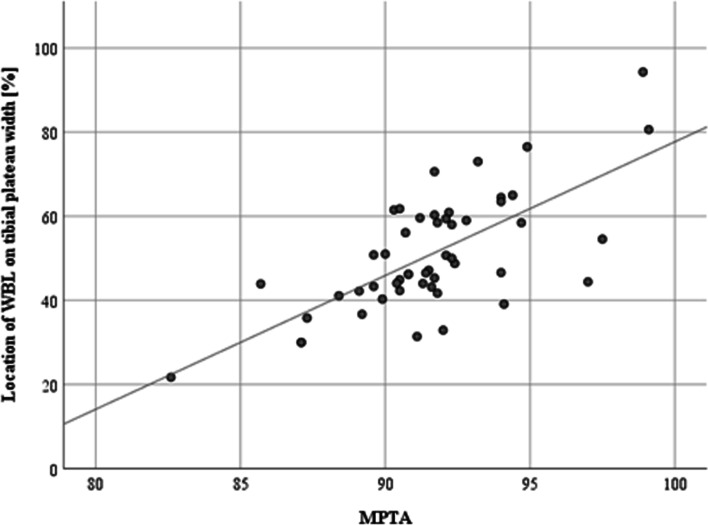
Linear regression analysis showed a statistically significant correlation of the postoperative medial proximal tibial angle (MPTA) and the postoperative location of the WBL on the tibial plateau width ratio. An increase of the MPTA by one degree will lead to lateral shift of the WBL by 3.2% along the coronal tibial plateau width

Regarding the medial proximal tibial angle (MPTA) no significant differences between both groups were observed preoperatively. However, significant differences of the MPTA were revealed after the operative procedure with the Conventional-Group presenting statistically significant higher values of the MPTA (*p* = 0.04) (Table 6). Linear regression analysis showed a statistically significant correlation between the postoperatively achieved MPTA and the WBL after HTO procedure (*p* = 0.00). An increase of the MPTA of 1° will lead to a lateral shift of the WBL of 3.2% along the coronal tibial width (Fig. 3).

**Table 6:** MPTA for the Noyes- and Conventional-Group

Variable	Noyes-Group	Conventional-Group	***P***-value (comparison between both groups)
**MPTA preoperative (mean ± SD)**	84.4° ± 2.7°	84.5° ± 2.6°	0.89
**MPTA postoperative (mean ± SD)**	90.7° ± 2.3°	92.4 ± 3.4°	0.04*
***P*-value (comparison within a group)**	0.00*	0.00*	

Preoperative and postoperative mean values of the medial proximal tibial angle (MPTA) for the Noyes- and Conventional-Group respectively. *P*-values are displayed for comparison of the mean MPTA from the pre- to postoperative visit within a group (last row) as well as for comparison of the MPTA between groups (last column). Statistically significant values are marked by asterisks

The Joint line convergence angle (JLCA) did not differ significantly before and after the operative procedure between both groups. However, within the Conventional-Group a significant decrease of the JLCA was noted from the preoperative to the postoperative visit (*p* = 0.03) (Table 7).

**Table 7:** JLCA for the Noyes- and Conventional-Group

Variable	Noyes-Group	Conventional-Group	***P***-value (comparison between both groups)
**JLCA preoperative (mean ± SD)**	1.8° ± 1.3°	2.5° ± 1.8°	0.10
**JLCA postoperative (mean ± SD)**	1.8° ± 1.3°	1.9° ± 1.4°	0.85
***P*-value (comparison within a group)**	0.98	0.03*	

Preoperative and postoperative mean values of the joint line convergence angle (JLCA) for the Noyes- and Conventional-Group respectively. *P*-values are displayed for comparison of the mean JLCA from the pre- to postoperative visit within a group (last row) as well as for comparison of the JLCA between the groups (last column). Statistically significant values are marked by asterisks

## Discussion

Accurate alignment correction according to the preoperatively determined parameters is a key factor for successful outcome of the HTO procedure. This study demonstrated that with the proposed method by Noyes the PTS remains mainly unchanged while achieving similar alignment correction in the coronal plane compared to the conventional method. This can be considered a major finding as unintended changes of the PTS during the HTO are generally believed to alter the natural knee kinematics. This is the first study to examine clinical data regarding the mathematically derived approach by Noyes.

Several studies have demonstrated an increase of the posterior tibial slope with the procedure of a medial opening wedge HTO [5, 7, 20]. However, most of the time when performing a valgus producing tibial osteotomy alterations of the sagittal plane are not intended and are primarily seen as an unintentional side effect of the coronal valgus correction. While the extend of the modification to the sagittal plane is usually subtle with a reported increase by 2 to 4° on the tibial slope, the influence of these modifications on the ligamentous intact knee are generally believed to be of minor importance. Nonetheless, an increased posterior tibial slope does alter knee kinematics to some degree. Firstly, in an ACL-deficient knee an intensified anterior tibial translation may be seen. Secondly, the increased anterior tibial translation leads to a shift of the tibiofemoral contact area redistributing the contact pressure into the posterior tibial plateau which may contribute to a premature failure of the HTO procedure [7, 11, 14, 20]. Another problematic issue with an increased PTS lies within hampered total knee extension, which is already reduced in an arthritic knee joint prior to surgery. Thus, besides rearranging the coronal alignment through the high tibial osteotomy, another key objective is to preserve the posterior tibial slope during the operative procedure. Interestingly, data of this retrospective study have shown that the posterior tibial slope was not altered significantly from the pre- to the postoperative visit when the opening wedge osteotomy was performed based on the mathematical model of Noyes et al. (Noyes-Group). However, there was a statistically significant increase of the PTS when the conventional method of intraoperative coronal alignment assessment was performed (Conventional-Group). Postoperatively, the PTS also turned out to be significantly higher in the Conventional-Group. This finding suggests that by the proposed method of Noyes et al. the sagittal plane seems to be influenced to a remarkedly lesser degree than when using the conventional method. Regarding the correction of the coronal alignment, no statistical difference was observed and the number of patients corrected within the accepted range of accuracy did not differ significantly between both groups. Nevertheless, undercorrection in the coronal plane is a frequently encountered problem in opening wedge HTO [1, 16, 23]. This factor also applies to the present study. Postoperatively, the mean WBL ranged at 50% from the medial to lateral tibial plateau width for both groups, compared to planned 62.5%. Though not being statistically significant, it seems noteworthy that not a single patient was overcorrected in the Noyes-Group compared to 11.5% in the Conventional-Group. These findings match the results of this study concerning the JLCA: For the Noyes-Group the change of the JLCA from the preoperative to the postoperative visit was not statistically significant, only revealing a minor decrease in the mean JLCA. In the Conventional-Group, the change of the JLCA turned out to be larger with a statistically significant decrease of the mean JLCA from the preoperative to the postoperative visit. A recent study by Lee et al. demonstrated that increasing changes of the JLCA were associated with greater over-correction of the lower limb alignment [15]. This seems to be a noteworthy finding, since data of the present study suggests that the Noyes method influences the JLCA to a lesser degree. Hence, coronal realignment by the Noyes method might be the preferred method for treatment of double varus knees with separation of the lateral tibio-femoral compartment, as higher changes of the JLCA are usually encountered in knees with a high degree of soft tissue laxity, often resulting in postoperative alignment overcorrection [17]. However, further research on this topic needs to be performed. With regard to the bony correction of the tibia a more anatomic postoperative MPTA was achieved in the Noyes-Group compared to a slight overcorrection of the MPTA in the Conventional-Group. Data from this retrospective analysis suggest that the Noyes approach may lead to fewer coronal bony corrections as measured by the MPTA compared to the conventional approach. This seems to be an important finding, as the change of the MPTA significantly correlates with the postoperative WBL ratio of the tibial plateau. In our current study we demonstrated that with a mean increase of the MPTA by one degree, the WBL ratio is raised by 3.2%.

We are aware that the current study has several limitations. As this research was designed as a retrospective analysis, the level of evidence should generally be considered inferior to prospective cohort studies and results may be subject to bias. Furthermore, as the postoperative radiographs were usually obtained 6 weeks postoperatively, radiographic data of this study should generally be considered as short-time result following the operative procedure. Due to inconsistent follow-up records adverse events like recurrent varus alignment or a conversion to total knee arthroplasty could not be elucidated adequately. Furthermore, correlation of this radiographic data with clinical outcome scores would have added additional value to this study. Yet patient number of this study seemed to be sufficient compared to similar retrospective analyses. Moreover, potential bias was limited by nearest neighbor matching of the data. This is the first study reporting real life clinical data of the Noyes method for high tibial valgus osteotomies, thus representing a valuable reference point for further research regarding this topic.

## Conclusion

Based on the results of this study the 3-trianglel method by Noyes may be a promising approach to preserve the posterior tibial slope during HTO.

## Abbreviations

ACL: Anterior cruciate ligament
HTO: High tibial osteotomy
JLCA: Joint line convergence angle
mLDFA: Mechanical lateral distal femur angle
MPTA: Medial proximal tibial angle
PACS: Picture archiving and communication system
PTS: Posterior tibial slope
TKA: Total knee arthroplasty
WBL: Weight bearing line
